# Breaking Barriers in Alzheimer’s: Next‐Gen Small Molecules Targeting USP14 for Neuroprotection and Protein Homeostasis

**DOI:** 10.1002/alz70859_106289

**Published:** 2025-12-25

**Authors:** Ajish Ariyath, Prashant Bharadwaj, Warnakulasuriya Mary Ann Dipika Binosha Fernando, Ralph N Martins

**Affiliations:** ^1^ Edith Cowan University, Perth, Western Australia Australia; ^2^ Edith cowan university, perth Australia

## Abstract

**Background:**

In Alzheimer's disease (AD) brains, autophagy is over activated, however emerging research suggests that the ubiquitin‐proteasome system might clear aggregation proteins and be a therapeutic target for AD and protein misfolding diseases. This study developed and validated novel small compounds targeting USP14 to improve protein homeostasis, reduce Aβ toxicity, and promote proteostasis in AD cell and worm models.

**Method:**

Using the MC65 AD cell model, we screened 71 small molecule proteostasis modulators, identifying two promising USP14 ligands, AA10 and AA51. Their effects on autophagy, proteasome activity, Aβ toxicity, neurodegeneration, and behavior were assessed via fluorescence microscopy, toxicity analysis, western blotting, behavioral, and lifespan analysis in *C. elegans*.

**Result:**

The AI‐driven drug discovery platform AtomNet enabled the design and screening of 72 novel USP14‐binding ligands. Two promising USP14 ligands AA10 and AA51 were tested in *C. elegans* AD model, neurodegeneration was reduced by 77% (IU1), 83% (AA10), and 84% (AA51) compared to control 33%. Furthermore, treated worms exhibited reduced lysosomal dysfunction through autophagic pathway modulation, enhanced behavioral activity (IU1‐83%, AA10‐88%, AA51‐83%) compared to control 47%, and extended lifespan (IU1‐4days, AA10‐5days, AA51‐5days), reinforcing their therapeutic potential. Notably, the use of autophagy inhibitors such as Bafilomycin A (BafA) and Chloroquine provided protection against Aβ toxicity, improved lifespan and behavior in AD worm model.

**Conclusion:**

This is the first report of IU1’s use in AD models as a USP14 inhibitor. AA10 and AA51 showed superior efficacy, making them promising candidates for future preclinical validations. The next phase involves testing therapeutic efficacy and brain bioavailability in AD mouse models to advance their potential as future treatments.

